# High Quality Draft Genome of Arogyapacha (*Trichopus zeylanicus*), an Important Medicinal Plant Endemic to Western Ghats of India

**DOI:** 10.1534/g3.119.400164

**Published:** 2019-06-12

**Authors:** Biju Vadakkemukadiyil Chellappan, Shidhi PR, Sheethal Vijayan, Veena S. Rajan, Anu Sasi, Achuthsankar S. Nair

**Affiliations:** Department of Computational Biology and Bioinformatics, University of Kerala, Thiruvananthapuram, Kerala, India

**Keywords:** Trichopus zeylanicus, Arogyapacha, Secondary metabolic pathways

## Abstract

Arogyapacha, the local name of *Trichopus zeylanicus*, is a rare, indigenous medicinal plant of India. This plant is famous for its traditional use as an instant energy stimulant. So far, no genomic resource is available for this important plant and hence its metabolic pathways are poorly understood. Here, we report on a high-quality draft assembly of approximately 713.4 Mb genome of *T. zeylanicus*, first draft genome from the genus *Trichopus*. The assembly was generated in a hybrid approach using Illumina short-reads and Pacbio longer-reads. The total assembly comprised of 22601 scaffolds with an N50 value of 433.3 Kb. We predicted 34452 protein coding genes in *T. zeylanicus* genome and found that a significant portion of these predicted genes were associated with various secondary metabolite biosynthetic pathways. Comparative genome analysis revealed extensive gene collinearity between *T. zeylanicus* and its closely related plant species. The present genome and annotation data provide an essential resource to speed-up the research on secondary metabolism, breeding and molecular evolution of *T. zeylanicus.*

Western Ghats in India is a major repository of many important medicinal plants. Indigenous people traditionally use these plants for their primary health care. In fact, the knowledge accumulated by the local inhabitants on many of these medicinal plants are still unknown to the scientific world. *Trichopus zeylanicus* subsp. *travancoricus*, belongs to the family *Dioscoreaceae* (APG II, 2003) (The Angiosperm Phylogeny Group (2003)) is such a rare medicinal plant, indigenous to Western Ghats of India (Figure 1). The medicinal properties of this plant were unknown to the scientific community until a scientific expedition to this forest in 1987 (Pushpangadan P. (1988)). This plant is famous for its traditional use by the local tribal people (known as Kani tribes, settled in Agastya hills, Western Ghats, India) to combat fatigue (Pushpangadan P. (1988)). It is locally known as “Arogyapacha”, literally meaning “the green that gives strength”. *T. zeylanicus* gained a global attention because of the first benefit-sharing model with tribals, for commercialization (Pushpangadan *et al.* (2018)). Besides its anti-fatigue properties, this plant is also shown to possess a varied spectrum of pharmacological properties such as anti-oxidant (Sharma *et al.* (1989); Sindhu and Jose (2015)), antistress (Pushpangadan *et al.* (1995)), aphrodisiac (Subramoniam *et al.* (1997)), anti-microbial (Vignesh *et al.* (2016)), anti-inflammatory (R *et al.* (2012)), immunomodulatory (Rishikesh *et al.* (2017)), anti-tumor (Pushpangadan *et al.* (1995)), anti-ulcer (Rishikesh *et al.* (2017)), anti-hyperlipidemic (VishnuVardhan Reddy *et al.* (2014)), hepatoprotective and anti-diabetic (Sundar Rajan and Velmurugan (2015)). Even though phytochemical screening of different extracts have revealed the presence of secondary metabolites such as phenolic compounds, alkaloids, flavonoids, tannins, terpenoids, steroids glycosides and saponins in *T. zeylanicus*, so far only a small number of phytochemicals have been isolated from this plant (R *et al.* (2012); Sindhu and Jose (2015); Sundar Rajan and Velmurugan (2015)). This is either because of the low production of secondary metabolites during the isolation or the difficulty in deducing chromatographic peaks of novel compounds in mass spectrometry analysis. So far, no genome resources is available for *T. zeylanicus* which further hinders the speedy research on this important medicinal plant. To provide the genome resource for the research community and also to get better insight into the metabolic potential and decipher the key genes associated with the synthesis of secondary metabolites, we sequenced the whole genome of *T. zeylanicus* using an integrative approach combining both Illumina short-reads and Pacbio long-reads. The annotation of the genome has identified large number of genes associated with diverse potential secondary metabolic pathways in this plant. The present draft genome offer a valuable resource for the identification of important metabolites and genetic breeding in *T. zeylanicus.*

**Figure 1:**
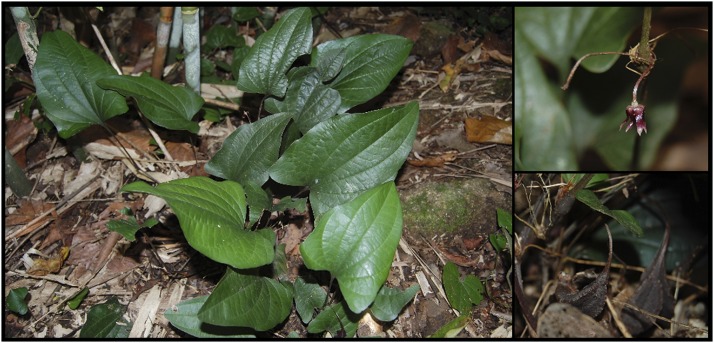
*Trichopus zeylanicus* (subsp. *travancoricus*). Left: Whole Plant Right above: Flower, Right below: Seeds.

## Materials and Methods

### Plant collection and genomic DNA isolation

The plant material was collected from Agastya hills, Trivandrum, Kerala (India). Genomic DNA was isolated from tender leaf tissues using CTAB method followed by 0.5× bead purification twice for both Illumina and Pacbio sequencing (Doyle and Doyle (1987)). The quality of the DNA sample was assessed using 0.75% agarose gel assay and Nanodrop (Nanodrop Technologies, Wilmington, DE, US), and was quantified using Qubit system (Thermo Fisher Scientific, Waltham, MA).

### Genome size estimation

Genome size of *T. zeylanicus* was determined by flow cytometry as described by Dolezel *et al.* (Doležel *et al.* (2007)). In brief, 100 mg of fresh leaves of *T. zeylanicus* subsp. *travancoricus* (2n = 28) (Ramachandran (1968)) and *Raphanus sativus* L. ‘Saxa’ (2n = 18), used here as a reference, were co-chopped in 1 ml ice cold Otto I solution (0.1 M citric acid (Sigma-Aldrich), 0.5% (vol/vol) Tween 20 (Sigma-Aldrich)) for one minute with a razor blade. After incubation for 2 min, the suspension was filtered through a 42-µm nylon mesh (Sigma-Aldrich) and centrifuged at 150g for 5 min at 40C. Supernatant up to approximately 100 µl of the liquid above the pellet was removed and the pellet was re-suspended in 100 µl Otto I solution. Prior to the analysis on flow cytometer, I ml of Otto II solution (0.4 M Na2HPO4 12H2O (Sigma-Aldrich) mixed with 50 µg of RNAse (Sigma-Aldrich) and propidium iodide (Sigma-Aldrich) was added to the nuclear suspension. After incubation for 5 min, over 5000 nuclei were passed through FACSAriaII system (BD Biosciences) to analyze the relative DNA content. Nuclear content (2C) of the sample was calculated as Sample 2C mean peak position/ Reference 2C mean peak position X Reference 2C value. The ratio of G1 peak mean of *T. zeylanicus* and *R. sativus* was equal to 1.57 and hence the 2C DNA amount of *T. zeylanicus* was estimated as 1·74 pg. 1C genome size of *T. zeylanicus* was estimated to be 860 Mb (1 pg = 0.978 X 109 bp) (Figure S1A) (Doležel *et al.* (2007)). In another method, the genome size was estimated based on number of reads and 21 K-mer frequency using Jellyfish version 2.0 on “clean” Illumina data from 3 insert library (Figure S1B) (Marçais and Kingsford (2011)). K-mer frequency showed a highest peak depth at 80 and total number of K-mers was 68197129747. Thus, the *T. zeylanicus* genome size was calculated to be approximately 852.4 Mb, using the formula: size = K-mer number/peak depth.

### Library preparation and sequencing

For Illumina sequencing, 1 µg high quality genomic DNA was fragmented using S220 Focused-ultrasonicator system (Covaris Inc, USA). Three paired end (PE) libraries were constructed from the fragmented DNA according to the standard protocol for the NEBNext Ultra II DNA Library Prep Kit for Illumina (New England Biolabs, Inc.) with average insert sizes of 300 bp, 500 bp and 800 bp. The quantity and quality of the library were assessed on TapeStation 4200 (Agilent Technologies, USA) using a high-sensitivity D5000 ScreenTape assay kit, as per the manufacturer’s instructions. After the library profile analysis, the PE libraries were sequenced using an Illumina HiSeq 2500 platform (2 X 100 bp) (Illumina Inc.). For Single Molecule Real Time (SMRT) sequencing, 30 µg of genomic DNA was mechanically sheared using Covaris g-TUBE (Covaris Inc, USA). SMRTbell templates were constructed from 5 µg sheared DNA using SMRTbellTM Template Prep Kit 1.0 0 (Pacific Biosciences, USA) following the manufacturer’s protocol. The templates were purified using AMPure PB beads (Pacific Biosciences, USA) and size ranging from 15 kb to 50 kb were selected using BluePippin (Sage Science, USA). Primer annealing and polymerase binding to SMRT template were conducted using DNA/Polymerase Binding Kit P6 v2 (Pacific Biosciences, USA). The DNA polymerase/template complexes was captured using The MagBead Kit v2 (Pacific Biosciences, USA) and were sequenced on Pacbio Sequel instrument with 5 SMRT cells using the P6 polymerase/C4 chemistry combination.

### Denovo hybrid assembly and quality assessment

Prior to assembling the genome, all raw reads were filtered and trimmed for any sequencing adapters, N’s at the end of the reads and low quality bases using AdapterRemoval2 (version 2.0) (Schubert *et al.* (2016)) (Table S1). Before the genome assembly, both Illumina and Pacbio total reads were mapped to chloroplast genome and mitochondrial genome of related plant species listed in Table S2 using BWA (version 0.7.9a-r786) (Langmead *et al.* (2009)) to extract the reads corresponding to *T. zeylanicus* chloroplast and mitochondrial genome, respectively. High quality (phred score <30) Illumina paired-end reads and Pacbio data were together used to construct a hybrid assembly. MaSURCA version 3.2.3 genome assembler was used for scaffolding and gap-filling (Zimin *et al.* (2013)). The completeness of the genome assembly was evaluated using BUSCO (Benchmarking Universal Single-Copy Orthologs) version 3.0.1 with 1440 conserved orthologous gene sets specific to plant from Embryophyta_odb9 (http://busco.ezlab.org/) (Simão *et al.* (2015). To further assess the quality and coverage of the assembly, the raw reads from three Illumina insert libraries were mapped back to the genome (Langmead *et al.* (2009)).

### Repeat identification

A *de novo* repeat library was made using RepeatModeler (version open-1.0.11) which was installed along with RECON v1.07, RepeatScout25 v1.0.5 and Tandem Repeat Finder (Tarailo-Graovac and Chen (2009)). The *de novo* repeat library from RepeatModeler was combined to Repbase repeat library (version 23.09) to annotate repeat regions in the assembly using RepeatMasker (version open-4.0.7) (Tarailo-Graovac and Chen (2009)).

### Protein coding gene prediction and functional annotation

Both homology and *de novo* based predictions were used to predict protein-coding regions in the genome of *T. zeylanicus*. In the homology based prediction, repeat-masked assembly was BlastX searched against NCBI non-redundant protein database with an e-value cut-off of 1e5. Protein sequences of significant hits were downloaded from the NCBI database and aligned to the assembly using Exonerate (version 2.2.0) to predict potential splice sites (Slater and Birney (2005)). In *de novo* method, genes were predicted on repeat-masked genome using two ab initio gene prediction tools Augustus (version 2.5.5) and Fgenesh of MolQuest (version v2.4.3.1111) by selecting *Zea mays* and *Dioscorea alata* as model organism, respectively (Stanke *et al.* (2006), Solovyev *et al.* (2006)). Finally, all gene prediction information was merged using EvidenceModeller (version v1.1.1) to generate a non-redundant gene set (Haas *et al.* (2008)). Gene models overlapped with transposable elements, with more than 50 N’s, coding sequence of length less than 150 bp were removed. Blast2Go (version 5.1.13) software was used for functional annotation. BlastP program incorporated in Blast2Go was used to search all protein sequences against Viridiplantae database (NCBI Non-redundant subset) to find homolog proteins (Conesa and Götz (2008)). InterProScan program incorporated in Blast2Go was employed to find conserved domain/motifs by searching the proteins against different functional domain databases such as Pfam, CDD, Panther, PIR and Coils (Zdobnov and Apweiler (2001)). Gene Ontology (GO) mapping was performed by Blast2Go. Two tailed Fisher’s exact test was performed for GO Enrichment analysis using the entire set of available *D. rotundata* proteins (BioProject: PRJDB3383) as a reference. The P value of Fisher’s exact test was set to P 0.001 to reduce the terms to most specific. For the identification of genes encoding disease resistant proteins in *T. zeylanicus*, complete protein sequences were searched (BlastP) against reference resistant proteins downloaded from Pathogen Receptor Genes database 3.0 (PRGdb 3.0). To identify the abundance of resistance genes in *T. zeylanicus* compared to its closely related species (*D. rotundata*, *Elaeis guineensis* (BioProject: PRJNA192219), *Ananas comosus* (BioProject: PRJNA305080) and *Asparagus officinalis* (BioProject: PRJNA376608)), resistance genes in these species were also predicted using the same method as described above. Trans-membrane helices in the proteins were predicted using TMHMM server 2.0. Genes encoding transcription factors (TF) in *T. zeylanicus* and its closely related species (*D. rotundata*, *A. comosus*, *A. officinalis* and *E. guieensis*) were identified by searching all the corresponding protein sequences against TF reference protein sequences retrieved from Plant Transcription Database Version v4.0. KEGG (Kyoto Encyclopedia of Genes and Genomes) pathway analysis was performed using Blast2Go to identify and functionally classify genes potentially involved in various metabolic pathways.

### Non-coding RNA annotation

Transfer RNA (tRNA) genes were identified using tRNAscan-SE version 1.23 (Lowe and Eddy (1997)). Ribosomal RNA (rRNA) genes were detected using RNAmmer version 1.2 (Lagesen *et al.* (2007)). Other non-coding RNA, including microRNA (miRNA) and small nuclear RNA (snRNA) were identified using infernal version 1.1.2 using the Rfam 12.1 database (Griffiths-Jones *et al.* (2003); Nawrocki and Eddy (2013)).

### Comparative gene families and phylogenetic analysis

For inter species comparison, the genome and protein sequences of *D. rotundata* (BioProject: PRJDB3383), *E. guineensis* (BioProject: PRJNA192219), *A. comosus* (BioProject: PRJNA305080) and *As.officinalis* (BioProject: PRJNA376608) were downloaded from NCBI database. Orthologous protein-coding gene clustering and analysis was performed using Orthovenn (Wang *et al.* (2015)). The proteins of single copy genes were aligned separately using MAFFT and all the alignments were concatenated to form a “supergene” alignment (Gupta *et al.* (2012)). The maximum likelihood tree was constructed using MEGA 7.0.26 and tested with 1000 bootstrap replicates (Kumar *et al.* (2015)). The Jones-Taylor-Thornton matrix-based model was selected as the amino acid substitution model, as predicted by jModelTest 2.1.1 based on Akaike’s Information Criterion (AIC) (Darriba *et al.* (2012)). The species divergence times were estimated based on the Bayesian method implemented in BEAST v1.10.1 with JTT substitution model, with a strict molecular clock and Coalescent: Constant size tree prior (Drummond *et al.* (2006)). The analysis was performed on the data set used in the ML analysis with previously published calibration times (divergence between *D. rotundata* and *A. officinalis* was 120 MYA (Mennes *et al.* (2013)). BEAST MCMC (Markov Chain Monte Carlo) simulation were ran for 10000000 generations (Whidden and Matsen (2015)). TreeAnnotator (version v1.6.1) software was used to annotate the phylogenetic results generated by BEAST and the FigTree (version v1.3.1) was used to visualize the BEAST MCC tree. The collinearity genes were identified using MCScanX with default parameters (match score, 50; match size, 5; gap penalty, –1; overlap window, 5; E-value, 1e-5)(Wang *et al.* (2012)). The dot plot option in MCScanX was used to visualize the collinearity.

### Data availability

The raw sequence data and genome assembly have been deposited at NCBI SRA and Genome under BioProject ID PRJNA484861. The genome assembly and the annotated genes are also available for downloading at https://genomevolution.org/coge/GenomeInfo.pl?gid=54631 and the functional annotation datasets are available for downloading at https://keralauniversity.ac.in/trichopus-zeylanicus. Supplemental material available at FigShare: https://doi.org/10.25387/g3.8114123.

## Results and Discussion

### Genome sequencing, hybrid assembly and evaluation

**Figure 2:**
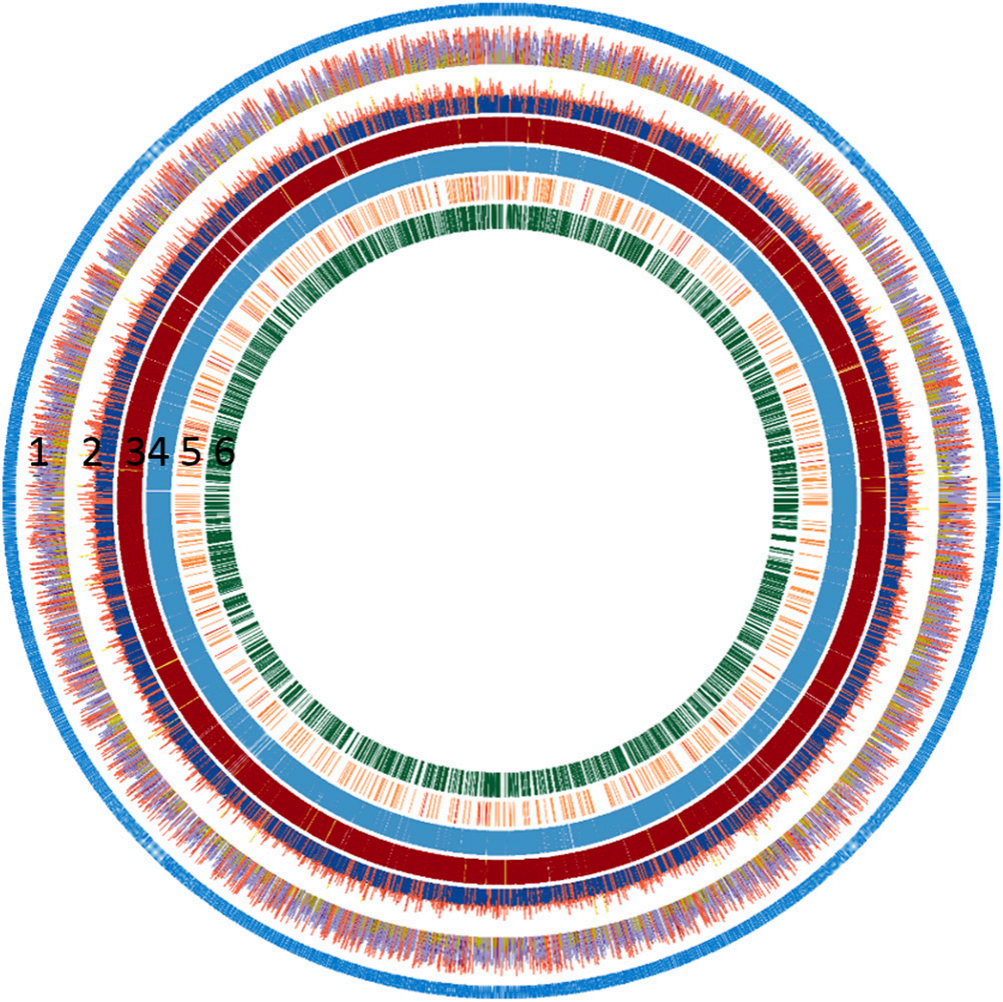
Genome characteristics of *T. zeylanicus*. 3433 Scaffolds (blue outer layer) of length >25 Kb were selected to schematically represent the genome of *T. zeylanicus* 1. Illumina read depth: Average read depth was calculated in a 25 Kb window size. Yellow indicates average depth <40, purple indicates average depth between 40 and 80 and red indicated average depth >80 2. GC content: Average GC content was calculated in a 25 Kb window size. Blue indicates average GC content less than 30, red indicated average GC content between 30 and 60 and yellow indicated average GC content >60.3. Transposable Elements: Yellow – Class II elements, red – Class I elements. 4. Simple repeats 5. Non-coding RNA: orange - mi-RNA, red – rRNA, green – snRNA, purple – tRNA 6. Non-TE genes.

We selected *T. zeylanicus* subsp. *travancoricus* (2n = 28), an endangered medicinal plant, endemic to Western Ghats of India for whole genome sequencing (Ramachandran (1968)) (Figure 1). The genome size was estimated to be 860 and 852 Mb according to flow cytometry and 21 K-mer distribution analysis (Figure S1). In total, 143.2 Gb of Illumina raw data were generated from 3 insert libraries of average size 300 bp, 500 bp and 800 bp (Table S1). After quality trimming, we obtained 136.5 Gb of high quality Illumina reads (Table S1). In addition, 17.5 Gb of longer reads with an average read length of 8 Kb were generated using Pacbio sequel platform. Prior to the genome assembly, all Illumina reads and Pacbio reads corresponding to chloroplast and mitochondria genome were removed that reduced the total size into 124 Gb and 14.9 Gb of Illumina and Pacbio reads, respectively. These data represented 144.1x and 17.3x coverage for the estimated genome size of *T. zeylanicus* (860 Mb). Both short reads and longer reads were used to construct a hybrid assembly using MaSURCA assembler (Zimin *et al.* (2013)). The initial assembly contained 25896 contigs of total length 712.6 Mb with a maximum contig length of 3.48 Mb. Total length of the final assembly (hereafter referred to as *T. zeylanicus* reference genome) after scaffolding was 713.4 Mb, distributed in 22601 scaffolds with maximum scaffold length of 3.9 Mb, which represents 83% of the estimated genome (860 Mb) (Table 1). The scaffold N50, N75 and N90 of the reference genome were 433 Kb (N50 index - 393), 111.3 Kb (N75 index - 1203) and 27.5 Kb (N90 - 3218), respectively. Of the total scaffolds, 2236 scaffolds contain gaps (3295 gaps) of total length of approximately 816 Kb with a maximum, minimum and average gap length of approximately 11.1 Kb, 20 bp and 247.7 bp, respectively. The evaluation of genome assembly using Benchmarking Universal Single-Copy Orthologs (BUSCO) (Simão *et al.* (2015)) revealed that 94.2% (1357 out of 1440 BUSCOs) of plant gene set were contained in our genome, demonstrating near complete representation of the genic space (Figure S2). To further evaluate and decipher the coverage for the reference genome, we mapped all high quality Illumina reads from three insert libraries to the scaffolds and we found that 867.9 million reads were remapped to the reference genome, of these, more than 84% were properly paired. The mapping of Illumina reads to the nuclear genome revealed an average of 80x read depth (Figure S1B, Figure 2). The scaffold N50 value (433 Kb) of *T. zeylanicus* genome assembly is small compared to that of the genome assembly of *D. rotundata* (scaffold N50: 2.1 Mb), *E. guineensis* (scaffold N50:1 Mb) and *A. comosus* (scaffold N50:11 Mb) (Table 1). The genomes of these species were scaffolded with genetic linkage maps into chromosome-scale assemblies, which the *T. zeylanicus* assembly is not (Table 1) (Singh *et al.* (2013); Ming *et al.* (2015); Tamiru *et al.* (2017)). Even though, our Scaffold N50 was short, contig N50 was remarkably longer (N50:288.8 Kb, L50:575) compared to that of genome assembly of *D. rotundata* (N50:18.8 Kb, L50), *E. guineensis* (N50:9.3 Kb, L50:28360) and *A. comosus* (N50:114.3, L50:834) (Table 1) (Singh *et al.* (2013); Ming *et al.* (2015); Tamiru *et al.* (2017)). Overall, the assembly generated in our study was a high quality draft (high contig N50, high average read depth, more than 84% of the estimated genome size, presence of most of the BUSCOs) genome of *T. zeylanicus* and addition of Hi-C sequence data would improve the present *T. zeylanicus* genome assembly into a chromosome-scale level.

**Table 1:** Statistics of genome assembly of *T. zeylanicus*

Feature	*T. zeylanicus*	*D. rotundata*	*E. guineensis*	*A. comosus*
BioProject	PRJNA484861	PRJDB3383	PRJNA192219	PRJNA305080
**Genome**				
Total length (Mb)	713.9	594.23	1535.17	382
GC content (%)	36.5	35.83	37	38.5
Total scaffolds (N)	22601	4723	40360	3129
scaffold gap size (Mb)	0.97	90	478	6.7
Scaffold N50 (Mb)	0.43	2.1	1	11.7
Scaffold L50 (N)	393	73	282	13
Total contigs (N)	25896	55363	1309411	9391
contigs N50 (Kb)	289.5	18.8	9.3	114.3
Contig L50 (N)	574	7420	28360	834
Repeat content (%)	47.4	46.15	57	51.8
LINE	0.05	2.4	1	1
Class I	34.2	22.8	47	31.6
Class II	1.8	6.7	2	4.1
Unknown	5.5	14.2	7	14.4
**Genes**				
Total predicted	34452	26198	41887	35775
GC content (%)	40.7	38.2	50	39
Total length (Mb)	86.5	125.5	263.5	132
Average length (bp)	2608	449	8587	4893
Number of Exons	155799	158059	339012	157953
Total Exon length (Mb)	34.5	42.4	98.8	40
Exon GC content (%)	49.3	44.08	45.23	49

### Repeats of T. zeylanicus genome

By combining both homology and *de novo* based approaches, we found that 47.4% of *T. zeylanicus *genome harbors repetitive elements which include both interspersed (295 Mb) and simple repeats (40.3 Mb) (Table 1, Table S3) (Figure 2). The repeat fraction is similar to that of *D. rotundata* (46.07%) but less than that in *E. guineensis* (57%) and *A. comosus* (51.8%) (Table 1) (Singh *et al.* (2013); Ming *et al.* (2015)). Among the interspersed repeats, Class I (Retrotransposons) were prominent (244.8 Mb) whereas Class II (DNA transposon) and Long Interspersed Nuclear elements (LINE) captured 104.5 Mb and 38.1 Mb of the genome, respectively (Table 1, Figure 2). The fraction of Class I elements (34.2%) in *T. zeylanicus* is similar to that of *A. comosus* (31.6%) but significantly abundant when compared to that of *D. rotundata* (22.8%) and significantly less when compared to that of *E. guineens* is (47%) (Table 1). The fraction of Class II elements in *T. zeylanicus* (1.8%) is comparable to that of *E. guineensis* (2%) but is less when compared to that of *D. rotundata* (6.7%) and *A. comosus* (4.1%). Within the Class I, Copia elements were most abundant (182.8 Mb) covering 25.7% of the genome, followed by Gypsi (51.1 Mb) (Table S3). We identified members of hAT, MuDR, PIF-Harbinger, EnSpm and Helitrons as the most abundant elements within the Class II (Table S3). In addition to the Class I and Class II elements, 5.5% of the genome contains repeats with no similarity to known elements and hence classified as unknown or lineage specific (Table 1). In total, 137106 simple sequence repeats (SSRs) were found, which constitutes 5.6% (40.3 Mb) of the genome (Nadeem *et al.* (2018)). Of these, tri-repeats were dominant (31% of the total number of SSRs), followed by hexa (21%), penta (13.9%), mono (8.3%), hepta (8.2%), tetra (7.7%) and di (6.9%). Overall, *T. zeylanicus* genome possessed high repeat content which might interfered the assembly of 16% of genomic region which is undetermined in our assembly.

### Gene prediction and functional annotation

**Figure 3:**
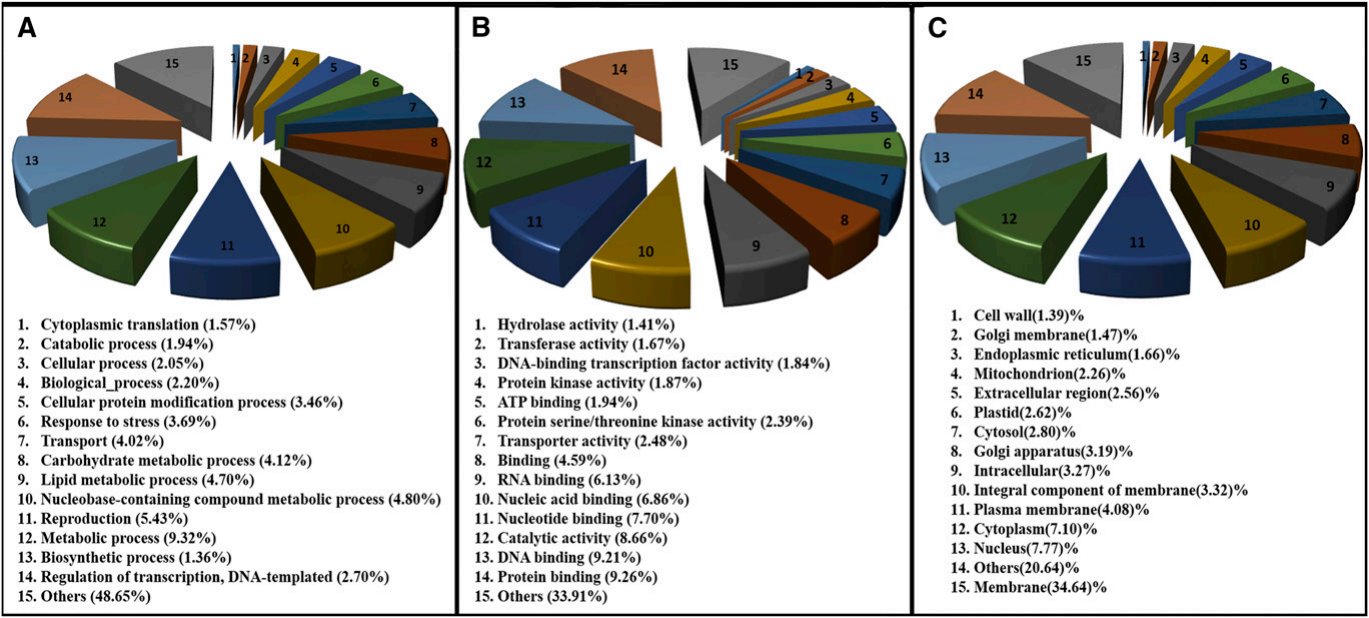
Gene ontology annotation of genes based on domains present in the encoded proteins. A. Biological Process classification. B. Molecular function classification C. Cellular component.

In sum, a set of 34452 protein-coding genes were predicted, which was comparable to that of closely related species (Table 1). The predicted genes were distributed on 2059 scaffolds with an average of 16.1 genes per scaffold (Table 1). In addition, we predicted 4547 non-coding RNA sequences, which included 3644 tRNA, 261 rRNA, 194 miRNA and 448 snRNA (Table S4). Of the 34452 protein-coding genes, 30090 (87.3%) proteins had significant similarity to known proteins in NCBI database and 28351 (82.2%) had InterProScan hit. Most represented protein domains were Protein kinase, Serine-threonine/tyrosine-protein kinase, Zinc finger, RING-type, RNA recognition motif domain and SANT/Myb domain (Table S5). Based on the sequence homology, gene ontology (GO) terms were assigned to 27282 genes which were further grouped into three major functional categories; biological process (3959 genes), molecular function (16879 genes) and cellular components (6443 genes). In biological process category, metabolic process (GO:0008152, 9.32%), reproduction (GO:0000003, 5.43%), nucleobase-containing compound metabolic process (GO:0006139, 4.80%), lipid metabolic process (GO:0006629, 4.70%) and carbohydrate metabolic process (GO:0005975, 4.12%) were prominent (Figure 3A). In molecular function category, protein binding (GO:0005515, 9.26%), DNA binding (GO:0003677, 9.21%), catalytic activity (GO:0003824, 8.66%), nucleotide binding (GO:0000166, 7.70%) and nucleic acid binding (GO:0003676, 6.86%) were overrepresented (Figure 3B). Similarly, in cellular component category, membrane (GO:0016020, 34.64%), nucleus (GO:0005634, 7.77%), cytoplasm (GO:0005737, 7.10%), plasma membrane (GO:0005886, 4.08%) and integral component of membrane (GO:0016021, 3.32%) were dominant (Figure 3C).

Gene ontology enrichment test for biological process revealed protein dephosphorylation (GO:0006470), translational initiation (GO:0006413), peptidyl-threonine phosphorylation (GO:0018107), glucose import (GO:0046323), seed trichome elongation (GO:0090378), tricarboxylic acid cycle (GO:0006099), glutathione metabolic process (GO:0006749), nucleosome assembly (GO:0006334), xyloglucan metabolic process (GO:0010411), activation of MAPKK activity (GO:0000186) and lignin catabolic process (GO:0046274) as enriched go terms (P value < 0.001) in *T. zeylanicus* compared to *D. rotundata* (Figure S3). Most of the GO terms associated with Molecular function including ATP binding, structural constituent of ribosome, heme binding, zinc ion binding were found to be significantly enriched (P value < 0.001) in *T. zeylanicus* compared to *D. rotundata* (Figure S3). cytosolic small ribosomal subunit, cytosolic large ribosomal subunit, SNARE complex and integral component of plasma membrane were found to be the most overrepresented GO terms associated with cellular components in *T. zeylanicus* compared to *D. rotundata* (Figure S3). Future gene expression and further follow-on experiments data are need to draw a conclusion about the biological relevance of these data.

### Genes associated with primary and secondary metabolic pathways

**Figure 4:**
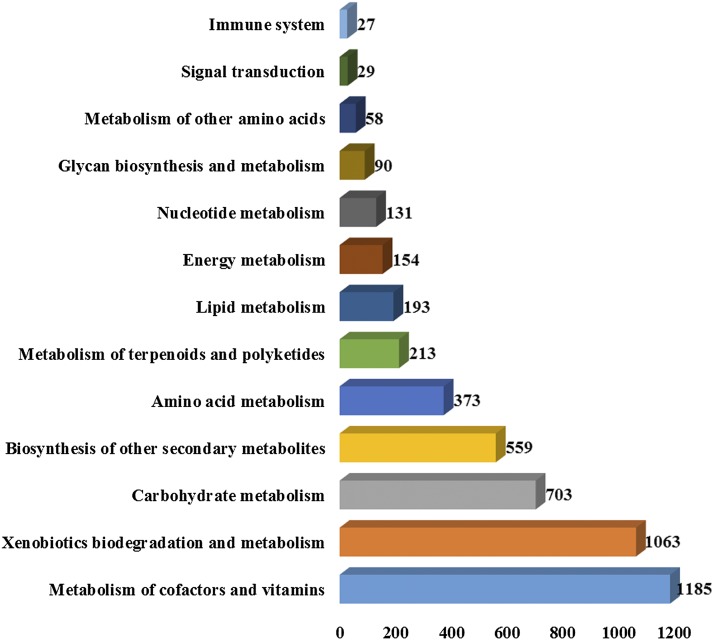
Major metabolic pathways identified in the genome of *T. zeylanicus* Number of genes associated with each pathway was shown adjacent to each bar.

To predict and functionally classify the genes potentially involved in various metabolic pathways, a KEGG (Kyoto Encyclopedia of Genes and Genomes) pathway analysis was performed using Blast2Go 5.1.13 software (Ogata *et al.* (1999)). In total, 4209 genes were initially mapped to 134 metabolic pathways. These pathways were further grouped into 15 major categories according to the canonical classes of the pathway maps in the KEGG database (Figure 4). Notably, of these 4209 genes 31.2% were assigned to 31 pathways involved in the synthesis of secondary metabolites. These pathways were further grouped into three major categories: (1) Metabolism of terpenoids and polyketides (11 pathways, 211 genes) (2) Xenobiotics biodegradation and metabolism (13 pathways, 1063 genes) (3) Biosynthesis of other secondary metabolites (18 pathways, 559 genes) (Table S6). The most five gene-enriched secondary metabolite pathways categorized in “Metabolism of terpenoids and polyketides” were terpenoid backbone biosynthesis, carotenoid biosynthesis, diterpenoid biosynthesis, zeatin biosynthesis and limonene and pinene degradation (Table S6). The most important pathways in “Xenobiotics biodegradation and metabolism” category were aminobenzoate degradation, drug metabolism - other enzymes, drug metabolism - cytochrome P450, metabolism of xenobiotics by cytochrome P450 and nitrotoluene degradation (Table S6). Similarly, Phenylpropanoid biosynthesis, caffeine metabolism, flavonoid biosynthesis, streptomycin biosynthesis and isoquinoline alkaloid biosynthesis were found to be the most gene-enriched pathways under “Biosynthesis of other secondary metabolites” category (Table S6). The number of genes and enzymes classes associated with the above mentioned secondary metabolite biosynthetic pathways were not significantly overrepresented in *T. zeylanicus* when compared to closely related plant species (Table S7). A future transcript level study combined with the present data and further biochemical characterization studies are required to reveal the importance of these genes in aforementioned pathways in *T. zeylanicus*.

### Genes encoding transcription factors

Since we found a significant portion of *T zeylanicus* genes were predicted to be involved in the secondary metabolite biosynthetic pathways, we, next, searched for genes encoding putative transcription factors because in many plant species, transcription factors play a major role in controlling the biosynthesis of secondary metabolites (Vom Endt *et al.* (2002)). We identified 1825 gene encoding transcription factors in the genome of *T. zeylanicus* and were classified into 51 families according to Plant Transcription Database at Centre for Bioinformatics, Peking University. Members of bHLH (182 genes) family were found to be most prominent, followed by 171 MYB, 141 C2H2, 137 NAC and 115 ERF (Table 2). The number of all these transcription factor families were significantly high in *T. zeylanicus* compared to that of *D. rotundata* (Table S8). The C2H2 family was found to be overrepresented in *T. zeylanicus* compared to *D. rotundata*, *A. comosus*, *E. guineensis* and *A. officinalis* (Table S8). C2H2 family is a large transcription factor family which is involve in normal plant growth and development as well as in many abiotic and biotic stress. Studies also showed that C2H2 transcription factors act as the positive regulators of many secondary metabolite biosynthesis (Ramamoorthy *et al.* (2013); Schoberle *et al.* (2014)). Further characterization of these genes, especially those related to secondary metabolism, is required to elucidate their role in *T. zeylanicus*.

**Table 2:** Transcription factor family in *Trichopus zeylanicus*

AP2 (31)	ARF (39)	B3 (31)	BBR-BPC (13)	BES1 (15)
bHLH (182)	bZIP (73)	C2H2 (141)	C3H (61)	CAMTA (7)
CO-like (19)	CPP (14)	DBB (7)	Dof (48)	E2F/DP (8)
EIL (14)	ERF (115)	FAR1 (26)	G2-like (42)	GATA (40)
GeBP (7)	GRAS (58)	GRF (13)	HB-other (6)	HB-PHD (4)
HD-ZIP (4)	HSF (23)	LBD (44)	LSD (4)	MIKC_MADS (29)
M-type_MADS (21)	MYB (171)	MYB_related (52)	NAC (137)	NF-X1 (1)
NF-YA (11)	NF-YB (17)	NF-YC (15)	Nin-like (3)	RAV (2)
SBP (26)	SRS (3)	TALE (23)	TCP (21)	Trihelix (34)
VOZ (43)	Whirly (2)	WOX (15)	WRKY (90)	YABBY (9)
ZF-HD (17)				

Note: Number of genes in each family is shown in brackets.

### Genes involved in Disease resistance

**Figure 5:**
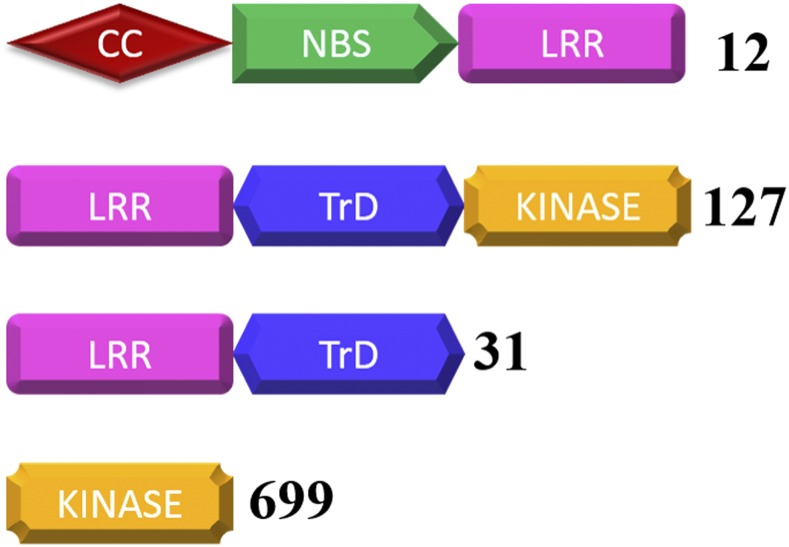
Schematic representation of resistant genes (R genes) classes in *T. zeylanicus* based on the arrangements of the functional domains. LRR - Leucine rich repeats; NBS -Nucleotide-binding site; CC - Coiled coil; TrD - Transmembrane domain, KINASE – Kinase domain. Number of genes in each classes is shown next to each figure.

We found that, among the 34452 protein sequences, 982 proteins possessed domains conserved in known plant resistant genes. Based on the domain organization, these proteins were classified into four major classes (Figure 5) (Hammond-Kosack and Jones (1997)). We identified 18 proteins with NB-LRR (Nucleotide-Binding Site-Leucine-Rich Repeat) domain and among these, six carried an additional CC (Coiled-Coiled) domains at their N terminal region (CC-NB-LRR) (Figure 5). Similarly, 127 and 31 proteins accounted for LRR-TrD-KINASE and LRR-TrD classes. The majority of predicted resistant proteins fall under the class “Enzymatic R genes” (699) (Hammond-Kosack and Jones (1997)) (Figure 5). Additionally, 58 and 68 gene were found in the genome with only NB domain and LRR domain, respectively, in their encoded proteins. Comparison to closely related species revealed that the number of resistance gene categories were less in *T. zeylanicus* (Figure S4).

### Comparative genomics and Phylogenetics

**Figure 6:**
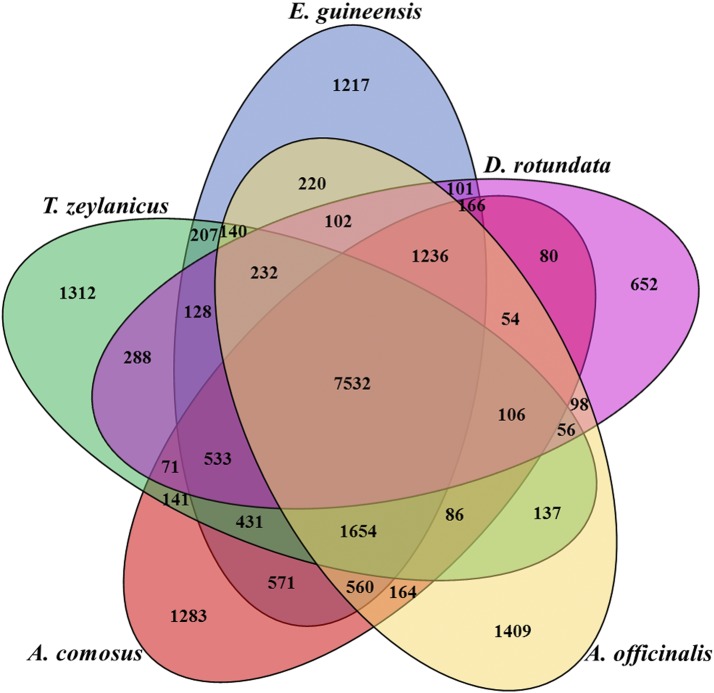
Orthologs genes shared among the monocot species. *T. zeylanicus* (TZ), *E. guineensis* (EG), *A. comosus* (AC), *D. rotundata* (DR) and *A. officinalis* (AO). Gene clustering was conducted using Orthovenn online tool.

**Figure 7:**
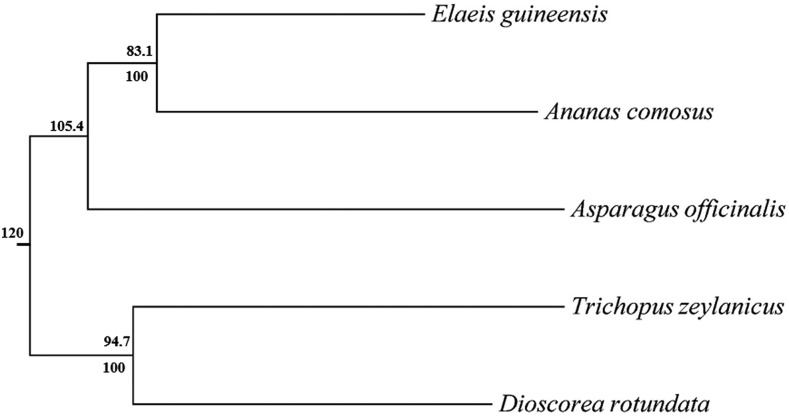
Phylogenetic tree and divergence time estimates. ML and Bayesian analysis based on protein alignment of 856 single copy genes in *Trichopus zeylanicus* and four other closely related species. Numbers above the nodes represent divergence times and below represent bootstrap values.

We examined orthologs of *T. zeylanicus* genes in four closely related species; *D. rotundata* (*Dioscoreaceae*), *A. officinalis* (*Asparagaceae*), *E. guineensis* (*Arecaceae*) and *A. comosus* (*Bromeliaceae*) using a reciprocal BlastP approach. About 46, 43, 47 and 40% of the genes in *T. zeylanicus* were orthologous to genes in *E. guineensis*, *A. officinalis*, *A. comosus* and *D. rotundata*, respectively. Based on the sequence similarity, the orthologs genes among these species were clustered into gene families. In total, 7532 gene clusters were shared by five species, among these 856 were single copy ortholog gene clusters. (Figure 6). Among these species, *T. zeylanicus* and *E. guineensis* shared more gene clusters (10440) compared to *T. zeylanicus* and *A. comosus* (10040), *T. zeylanicus* and *A. officinalis* (9409) and *T. zeylanicus* and *D. rotundata* (8888) (Figure 6). We found 12956 gene families in *T. zeylanicus*, among these, 1248 gene families appeared to be lineage specific (Figure 6). We constructed a maximum like-hood tree based on the protein alignment of 856 conserved single copy genes to infer the evolutionary relationship between *T. zeylanicus* and other four species. Within the tree, *T. zeylanicus* formed a clade with *D. rotundata* with 100% bootstrap value, confirmed their close relationship within the order *Dioscoreales* (family *Dioscoreaceae*) (Additional Figure 7). Based on the previously calibrated divergence time between *A. officinalis* and *D. rotundata* from their common ancestor (120 MYA), we estimated that *T. zeylannicus* diverged from *D. rotundata* about 94.7 MYA (Figure 7) (Mennes *et al.* (2013)). The synteny analysis revealed that *T. zeylanicus* and *E. guineensis* shared 1573 synteny blocks (a synteny block contains at least five reciprocal best-hit gene pairs) and 11384 collinear ortholog pair, *T. zeylanicus* and *A. comosus* shared 1316 synteny blocks and 9608 collinear ortholog pair and *A. officinalis* and *T. zeylanicus* shared 968 blocks and 6113 ortholog pair (Figure S5, S6, S7, Table S9A-C). Even though *T. zeylanicus* and *D. rotundata* shared 8888 gene clusters, we found only 193 synteny blocks and 1641 collinear genes (Figure S8, Table S9D). Further analysis of the *D. rotundata* chromosomal level assembly revealed that only 19023 genes were assigned to its pseudo chromosome and that reduced the collinearity between *T. zeylanicus* and *D. rotundata*. So we repeated the synteny analysis using scaffold level assembly of *D. rotundata* and found 1305 synteny blocks and 8627 ortholog pair between *D. rotundata* and *T. zeylanicus* (data not shown). However, we noticed that the shared collinearity blocks and ortholog genes between *T. zeylanicus* and *D. rotundata* (both are currently assigned to family *Dioscoreaceae*) were less compared to that of shared by *T. zeylanicus* and *E. guineensis* (both are belong to distinct taxonomical orders). Perhaps, some ancestral genes in *D. rotundata* might have deleted after its divergence from *T. zeylanicus*. But it is difficult to conclude this point with the present draft genome assembly of *T. zeylanicus*. Alternatively, more shared genes between *T. zeylanicus* and *E. guineensis* compared to that of between *T. zeylanicus* and *D. rotundata* could be explained by the quality of the assembly and annotation of the genomes that we have selected for the synteny analysis. A chromosome-scale level assembly of *T. zeylanicus* will enable clearer synteny block analysis.

As a conclusion, the present study described the first draft genome from the genus *Trichopus*. The high quality draft genome and annotation reported in this study will be a strong foundation to speed up the research on *T. zeylanicus* to understand its biochemical diversity and pharmaceutical qualities. We found that a significant portion of genes were associated with secondary metabolite pathways including flavonoid, isoquinoline alkaloid, phenylpropanoid and terpenoid backbone biosynthesis. A transcript level study combined with the present data can elucidate the potential candidate genes in these pathways. We showed that *T. zeylanicus* possess high level synteny to its related species. Further comparative analysis in this species can also reveal the mechanism underlying the evolution of species specific secondary metabolism and chemical diversity in these species. Moreover, we found that 5.5% of the genome were simple repeats. Simple sequence repeats (SSRs) are tandem repeats motif comprised of 1-6 nucleotides. Because of its higher polymorphism, SSR are widely used in genetic studies and breeding programs of various taxa. Further characterization of these repeats can be utilized to develop molecular markers in *T. zeylanicus* breeding programs.
